# mAb MDR1-modified chitosan nanoparticles overcome acquired EGFR-TKI resistance through two potential therapeutic targets modulation of MDR1 and autophagy

**DOI:** 10.1186/s12951-017-0302-5

**Published:** 2017-10-04

**Authors:** Yan Zheng, Chang Su, Liang Zhao, Yijie Shi

**Affiliations:** 1 School of Pharmacy, Jinzhou Medical University, Jinzhou, 121000 People’s Republic of China; 2 School of Veterinary Medicine, Jinzhou Medical University, Jinzhou, 121000 People’s Republic of China

**Keywords:** EGFR, Tyrosine kinase inhibitor, Nanoparticles, Gefitinib, Autophagy, Chloroquine

## Abstract

**Background:**

Tyrosine kinase inhibitors (TKIs) that act against the epithelial growth factor receptor (EGFR) were once widely used in chemotherapy for many human cancers. However, acquired chemoresistance occurred in almost all patients, limiting the clinical application of EGFR-TKI. Thus far, no effective methods existing can resolve this problem. Designing a therapeutic treatment with a specific multi-target profile has been regarded as a possible strategy to overcome acquired EGFR-TKI resistance.

**Methods:**

MDR1 antibody-modified chitosan nanoparticles loading gefitinib and autophagy inhibitor chloroquine were prepared by ionic crosslinking and electrostatic attracting method. MTT assay, flow cytometry analysis and western blot assay were all performed to confirm the effect of different formulations of gefitinib on the proliferation of SMMC-7721/gefitinib cells. The preparations demonstrated their multi-target potential to achieve both tumor-targeting selectivity and the desired antitumor effects by blocking cell-surface MDR1 and inhibiting autophagy.

**Results:**

mAb MDR1-modified CS NPs, when combined with the co-delivery of gefitinib and chloroquine, showed targeting and therapeutic potential on enhancing the delivery of anticancer drugs and inducing significant cell apoptosis against acquired EGFR-TKI resistance through the modulation of autophagy and while blocking the activity of the MDR1 receptor.

**Conclusions:**

A new approach to design an excellent nanoparticle drug-delivery system can overcome acquired EGFR-TKI resistance against various multiple antitumor targets.
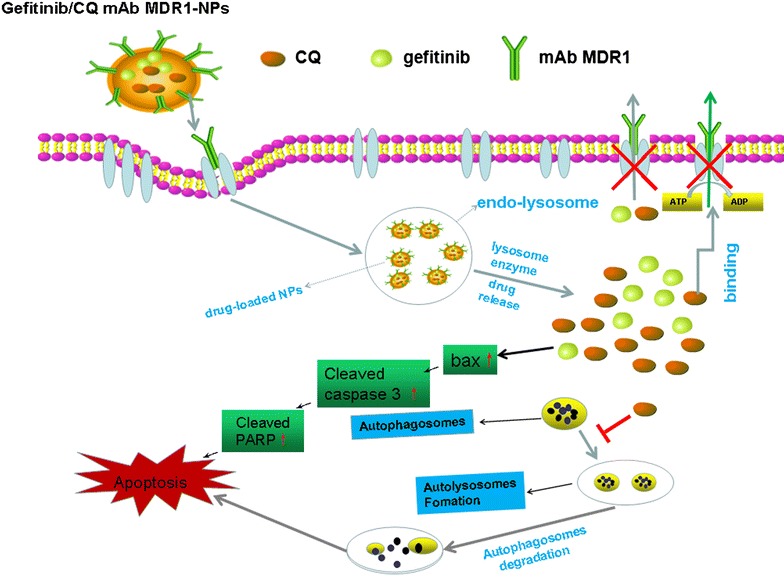

## Background

The epidermal growth factor receptor (EGFR) is a membrane-surface protein with tyrosine kinase activity. Studies have shown that it is highly expressed in most cancer patients, and that abnormal EGFR signaling pathways play an important role in tumorigenesis, tumor progression, and metastasis. Tyrosine kinase inhibitors (TKIs) that act against the EGFR (EGFR-TKIs), such as gefitinib, the first selective EGFR-TKI domain, can effectively prevent tumor growth, metastasis [1–3], and angiogenesis, and promote tumor cell apoptosis [4–6]. EGFR-TKIs are typically successful in the treatment of malignancies, especially for non-small cell lung cancer [7–10]. However, after a certain period of drug exposure, tumor cells gradually become insensitive to EGFR-TKIs, ultimately surviving following exposure to chemotherapy drugs. In this way, cells develop acquired chemoresistance, thus significantly reducing the therapeutic effect of EGFR-TKIs and limiting their clinical applications [11–14].

The occurrence of acquired resistance is very complicated and many reports demonstrate that the overexpression of MDR1 protein and the upregulation of autophagy are mainly attributed to acquired resistance. The MDR1 protein, also known as resistant protein, is primarily located in the cell membrane and its overexpression excretes extracellular chemotherapeutic drugs in tumor cells, resulting in reduced chemotherapeutic effects and insensitivity of drugs to tumor cells. Therefore, the inhibition of MDR1 could prevent the efflux of drugs and improve the efficacy of chemotherapy [15–19].

In autophagy, autophagosomes are lysed with lysosomes to form autolysosomes that degrade damaged and deformed macromolecules and organelles in the cytoplasm for normal cell survival. A large number of studies have shown that the augment of cell autophagy promoted tumor cell resistance and autophagy inhibition would be a potential target for reversing drug resistance [20–23]. HSF-1 upregulated Atg7 expression by directly binding to the ATG7 promoter which, in turn, activated autophagy and promoted tumor cell resistance [24]. Activation of reactive oxygen species (ROS)/ERK-mediated protective cell autophagy blocked the occurrence of apoptosis and ultimately led to tumor cell proliferation and a reduction in their sensitivity toward drugs [25].

Chitosan (CS) with the excellent biocompatibility, low toxicity and higher bioadhesion is a kind of natural cationic polymers, and especially suitable for building nanoparticle system to pass some molecules such as drug compounds, vaccines into cells. The cationic electricity allows CS to combine with some other functional substances having negatively charged ion and results in direct and effective delivery of drugs through the cell surface. Hence, we prepared CS nanoparticles (NPs) conjugated with the monoclonal antibody against MDR1 (mAb MDR1), which is capable of entrapping the anticancer drug, gefitinib, and chloroquine (CQ)—a known inhibitor of autophagolysosome formation—to explore whether EGFR-TKI resistance could be reversed in EGFR-TKI-resistant cancer cells. We used an excellent nanoparticulate drug-delivery system against multiple antitumor targets. The mAb MDR1 modified NPs loaded with gefitinib and CQ (gefitinib/CQ mAb MDR1-NPs) combined with MDR1 receptors which were situated at the surface of SMMC-7721/gefitinib cells (established gefitinib resistant) and they effectively enhanced drug accumulation in these cells, owing to the specific binding between mAb MDR1 and the MDR1 receptor. In addition, when compared with single-treatment therapy that targeting either MDR1 or autophagy, the combination of blocking MDR1 at the cell surface and inhibiting autophagy increased the intracellular accumulation of drugs and restored the cells’ sensitivity to the drugs, thereby reversing acquired EGFR-TKI resistance. Taken together, an excellent nanoparticulate drug-delivery system against multiple antitumor targets was a possible strategy to overcome acquired EGFR-TKI resistance.

## Methods

### Materials

Gefitinib was purchased from Eastbang Pharmaceutical Co., Ltd (Guangzhou, People’s Republic of China); Chloroquine, acetic acid and sodium tripolyphosphate (TPP) were obtained from Sigma (St Louis, USA). CS with the deacetylation degree of 80% and molecular weight of approximately 400 kDa was purchased from Haixin Biological Product Co., Ltd (Ningbo, People’s Republic of China). PBS and FBS were purchased from Thermo Fisher Scientific (Shanghai, China). Albumin Bovine V was got from Solarbio Technology Co., Ltd (Beijing, China) and Annexin V-FITC/PI Apoptosis Detection Kit was obtained from BestBio Technology Co., Ltd (Shanghai, China). The antibody used for the research such as MDR1/ABCB1 (E1Y7B) Rabbit mAb (mAb MDR1 we used), p Glycoprotein 1(MDR1), LC3A/B rabbit mAb, and cleaved-caspase3 Rabbit mAb were purchased from Cell Signaling Technology (Boston, USA) and bax, β-actin, cleaved-PARP, goat anti-rabbit lgG secondary antibody HRP were from AbSci (Maryland, USA). Human hepatocellular carcinoma cell lines SMMC-7721/gefitinib cells (established gefitinib resistant) were obtained from Jinzhou Medical University and were maintained at 37 °C in a humidified atmosphere of 5% CO_2_, 95% air. And other chemicals purchased such as MTT, Rhodamine B, sodium polyphosphate, SDS and glycine were of analytical grade and obtained from Sigma-Aldrich.

### Preparation and identification of gefitinib/CQ mAb MDR1-NPs

According to our previous reports [26, 27], CS NPs were prepared by an ionic crosslinking method. CS was dissolved in a solution of glacial acetic acid. When sodium polyphosphate (TPP) was added into the solution, positively charged CS was aggregated around the negatively charged TPP to form NPs. Once any residual was removed by washing the solution with distilled water and centrifugation, the prepared NPs were resuspended and free mAb MDR1 was added. As the CS NPs were positively charged and the mAb MDR1 was negatively charged, mAb MDR1 was modified to the NPs surface by electrostatic attraction. The obtained NPs were precipitated by centrifugation at 16,000 rpm for 20 min, then the NPs were separated and washed with PBS for three times to remove the free antibody. The green fluorescence emitted by mAb MDR1 was observed using a laser confocal microscope to determine whether the mAb MDR1 was conjugated on the surface of the CS NPs. The size, morphology, and zeta potential were characterized by transmission electron microscope (TEM) (JEM-1200EX; JEOL, Tokyo, Japan) and a Zetasizer (Nano ZS90; Malvern Instruments, Malvern, UK). The encapsulation ratios of gefitinib and CQ were measured with an ultraviolet–visible (UV–Vis) spectrometer and the in vitro drug-release behavior in the NPs was studied using a dialysis method with a Mw cutoff 1000, and each experiment was replicated for 3 times.

### Distribution and the cellular uptake of NPs

According to our previous reports [28], before the preparation of NPs, Rhodamine B (RhoB) was dissolved in the CS solution, and with the addition of TPP, RhoB was encapsulated in the core of the NPs to label their traction. A drug-resistant cell line SMMC-7721/gefitinib in a logarithmic growth cycle was seeded into the confocal dishes to reach a cell density of 5 × 10^5^ cells/mL. RhoB-labeled NPs and RhoB-labeled mAb MDR1 NPs were passed through a 0.22 μm filter and added into the confocal dish, and the SMMC-7721/gefitinib cells were incubated with the NPs. The location and distribution of RhoB-labeled NPs in the cells were visualized by confocal laser scanning microscopy (FluoView FV10i; Olympus Corporation, Tokyo, Japan) at given time intervals. The relative fluorescence ratio of NPs (RFR, %) was quantified by calculating the percentage ratio of fluorescence intensity emitted by internalized RhoB-labeled NPs cells to the initial fluorescence intensity from the total added RhoB-labeled NPs. And the process was: cells in full growth media were seeded in a 96-well plate (5 × 10^4^ cells/well) followed by the addition of RhoB-labeled NPs. The fluorescence intensity of the initially added RhoB-labeled NPs was determined by checking the fluorescence intensity of RhoB in each well. At different time interval, cold PBS was used to wash cells to remove the uninternalized NPs, while quantification of intracellular NPs was detected using a microplate reader (Synery-2; BioTek Instruments) by checking the fluorescence intensity of RhoB.

An endocytosis inhibition test was also performed by preincubating the tumor cells with chlorpromazine, genistein, cytochalasin D, and sodium azide for 2 h, followed by the treatment of RhoB-labeled NPs for continuous incubation. The relative uptake ratio was determined by comparing the relative fluorescence ratio of NPs treated with inhibitors with the relative fluorescence ratio of NPs treated with non-inhibitors.

### MTT assay

Cells were harvested by trypsin digestion and adjusted to 5 × 10^5^ cells/mL, and they were uniformly added into 96-well plates at a concentration of 100 μL cells per well and placed in a cell incubator for 24 h until they were adhered for extension. After that, according to the protocol of our previous study [28], free gefitinib; gefitinib and mAb MDR1; gefitinib and CQ; a mixture of gefitinib, CQ, and mAb MDR1; gefitinib NPs; gefitinib/CQ NPs; gefitinib mAb MDR1-NPs and gefitinib/CQ mAb MDR1-NPs, using the same gradient concentration of gefitinib that of 5, 10, 15, 30, 40 μg/mL, were used to treat SMMC-7721/Gefitinib cells for 24 h at 37 °C for further analysis. In all, 10 µL of the 12 mM MTT stock solution was added to each well and incubated for 6 h at 37 °C under 5% CO_2_ and 95% O_2_. Then, 50 µL of dimethyl sulfoxide was added into each well and mixed thoroughly with a pipette and incubated at 37 °C for 10 min. The absorbance of the solution was quantified using a BioTek Synergy-2 microplate reader to measure absorbance at 490 nm.

### Cell apoptosis evaluated by flow cytometry

An Annexin V (AV)–FITC/propidium iodide (PI) staining assay was performed, and apoptotic and necrotic cells were quantified by flow cytometry. According to the kit introduction and the protocol of our previous study [28], free gefitinib; gefitinib and mAb MDR1; gefitinib and CQ; a mixture of gefitinib, CQ, and mAb MDR1; gefitinib NPs; gefitinib/CQ NPs; gefitinib mAb MDR1-NPs and gefitinib/CQ mAb MDR1-NPs, featuring the same concentration of gefitinib that of 25 μg/mL, were used to treat SMMC-7721/gefitinib cells for 24 h at 37 °C for further analysis.

### Western blot assay

To further evaluate the cell apoptosis effects induced by free drug and drug-loaded NPs with the same concentration of gefitinib, western blot was used as a widely employed analytical technique to detect the expression levels of related proteins. Proteins in the cells were solubilized by employing buffers to promote cell lysis; the proteins were subsequently separated using gel electrophoresis. Further, proteins were transferred from the gel onto a membrane made of nitrocellulose or polyvinylidene difluoride (PVDF) followed by the blocking of non-specific binding. The film was put into the small box, and 15 mL of 1% BSA was added for the continous incubation for 90 min on the shaking bed. After incubation with a primary antibody and a secondary antibody, enhanced chemiluminescence was stained and the levels of the targeted proteins were photographed and analyzed using a UVP gel analysis system (iBox Scientia 600; UVP, LLC, Upland, CA, USA).

## Results

### Preparation and determination of the characteristics of gefitinib/CQ mAb MDR1-NPs

**Fig. 1:**
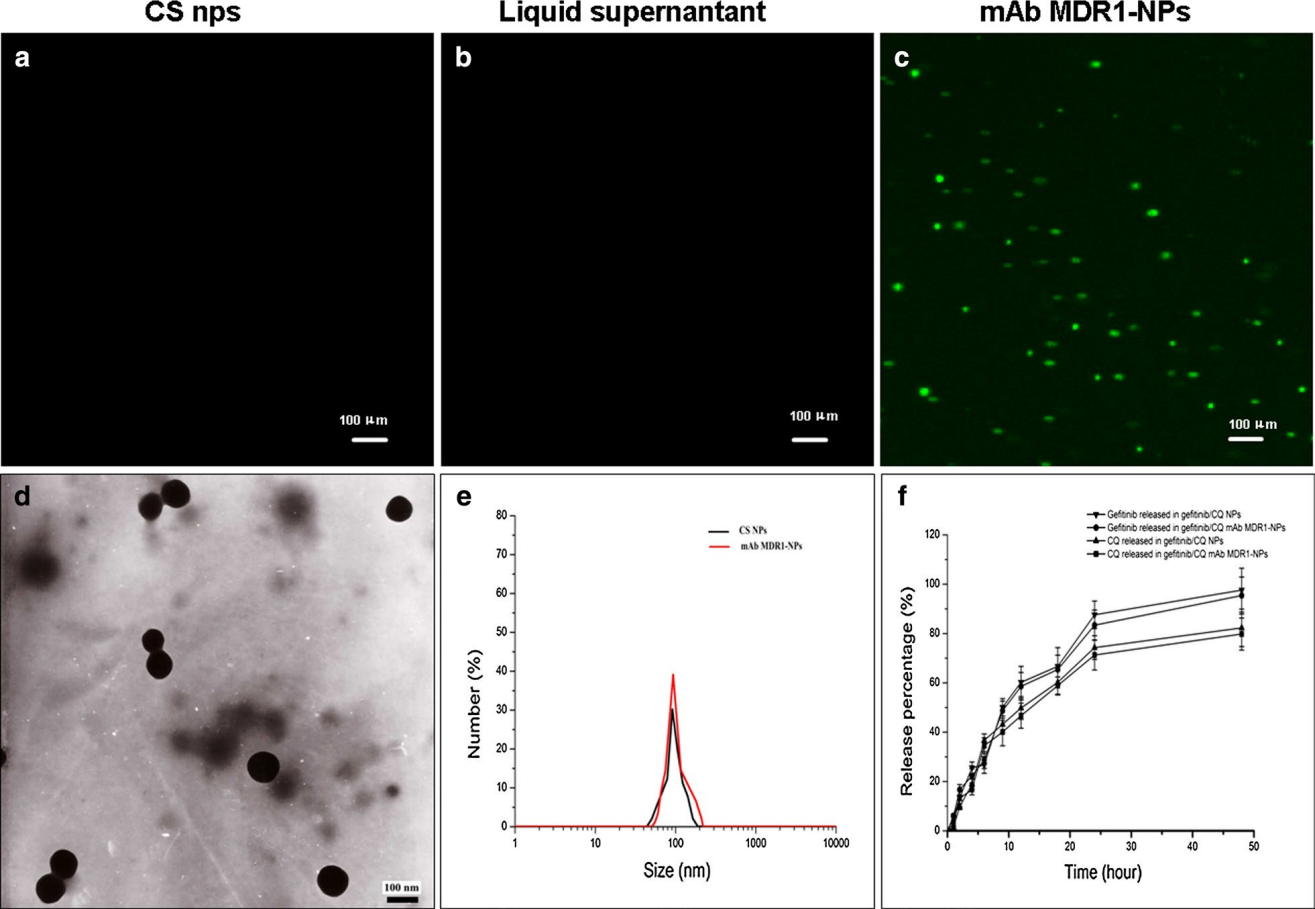
Fluorescence microscope images of identification of mAb MDR1 with immunofluorescence: **a** CS NPs; **b** liquid supernatant; **c** mAb MDR1-NPs. Characterization of gefitinib/CQ mAb MDR1-NPs: **d** TEM image of gefitinib/CQ mAb MDR1-NPs; **e** DLS analysis of gefitinib/CQ mAb MDR1-NPs; **f** accumulated release of gefitinib and CQ from gefitinib/CQ mAb MDR1-NPs in the medium at pH 7.4. The results are expressed as the mean ± SD (n = 3)

To justify the presence of mAb MDR1 at the surface of the CS NPs, we observed the green fluorescence emitted by mAb MDR1 to label the conjugation of mAb MDR1 with CS NPs. The CS NPs and mAb MDR1-NPs were dropped on the slides and observed under a fluorescent microscope (DMI4000B; Leica Microsystems GmbH, Wetzlar, Germany). As shown in Fig. 1, no fluorescent dots were found in the liquid supernatant, indicating that free green fluorescent mAb MDR1 had been completely removed from the surface of the mAb MDR1-NPs. Obvious green fluorescence was observed in the mAb MDR1-NPs, suggesting that the green fluorescent mAb MDR1 was successfully modified at the surface of the CS NPs to prepare the mAb MDR1-NPs; conversely, no obvious fluorescence was observed in the CS NPs.

The physical and chemical properties of gefitinib/CQ mAb MDR1-NPs, such as particle size, appearance, encapsulation efficiency, and in vitro release, were investigated. The results show that mAb MDR1-NPs exhibited a spherical morphology with a narrow size distribution. The average particle size of the CS NPs and the mAb MDR1-NPs was valued at 96.3 ± 6.3 and 103.8 ± 11.3 nm, respectively. The average zeta potentials were 22.6 ± 4.4 and 15.4 ± 6.4 mV, respectively. The encapsulation efficiency of gefitinib and CQ in mAb MDR1-NPs was 85.6% ± 9.2% and 88.7% ± 5.8%, respectively. The polydispersity index of the mAb MDR1-NPs was at about 0.14 ± 0.06. The in vitro release of gefitinib and CQ in both the CS NPs and mAb MDR1-NPs, as conducted by the dialysis bag method, showed a similar biphasic release. The accumulative release rates of gefitinib in both CS NPs and mAb MDR1-NPs had increased gradually from approximately 25.6 and 22.3%, respectively, in the initial 4 h to over 87.6 and 83.4% at 24 h and, finally, they were slightly enhanced to 97.6 and 95.4% at 48 h. The CQ encapsulated in the NPs depended on the variation of time required to control the release. The in vitro release percentage of CQ in the CS NPs and mAb MDR1-NPs were 18.9 and 16.7%, respectively, within the first 4 h, and about 74.3 and 71.3% of CQ were completely released within 24 h. Finally, more than approximately 82.3 and 79.8% of the total CQ slowly leaked out from the CS NPs and mAb MDR1-NPs into the medium within 48 h.

### Distribution and the cellular uptake of NPs

**Fig. 2:**
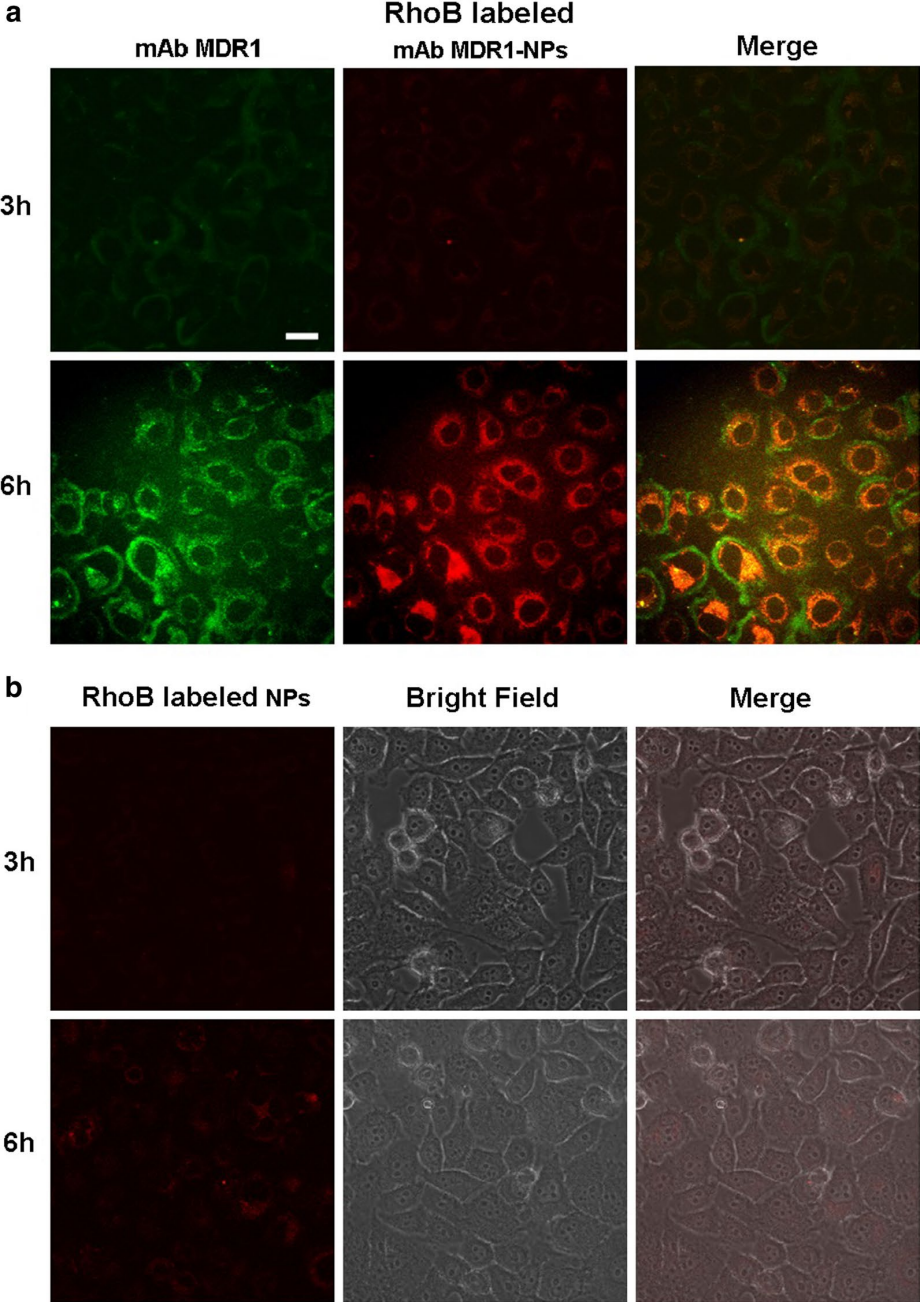
The in vitro cellular distribution of RhoB-labeled mAb MDR1-NPs and RhoB-labeled unmodified NPs following the incubation of SMMC-7721/gefitinib cells. **a** Fluorescent images of SMMC-7721/gefitinib cells incubated with RhoB-labeled mAb MDR1-NPs. **b** Fluorescent images of SMMC-7721/gefitinib cells incubated with RhoB-labeled unmodified-NPs. The scale bar in all figure parts is 100 μm

From Fig. 2, we found that RhoB-labeled mAb MDR1-NPs significantly enhanced the internalization of NPs into the cells compared with RhoB-labeled NPs. When the cells were co-incubated with mAb MDR1-NPs for 3 h, a small amount of red spots was concentrated and distributed around the cell membrane in the first 3 h (Fig. 2a). In addition, the co-localization between the green fluorescence emitted by mAb MDR1 and the red fluorescence from the RhoB-labeled mAb MDR1-NPs was obviously observed, suggesting that as the MDR1 antibody immediately bound to the MDR1 receptor (mAb MDR1 itself with green fluorescence) on the cell surface, mAb MDR1-NPs had aggregated around the cells, thus promoting the internalization of the NPs. With the passage of time, the red fluorescence intensity inside the cells was significantly enhanced, indicating that the NP uptake involved time-dependent internalization, and that more NPs were internalized and spread into the cytoplasm. It also signified that mAb MDR1-NPs effectively enhanced the drug accumulation within the cell surface of MDR1-overexpressed gefitinib-resistant cells. Furthermore, by blocking the MDR1 receptor with mAb MDR1, the sensitivity of gefitinib to gefitinib-resistant cells was enhanced, thus reversing the acquired resistance.

### Quantitative analysis of the cellular uptake of NPs

**Fig. 3:**
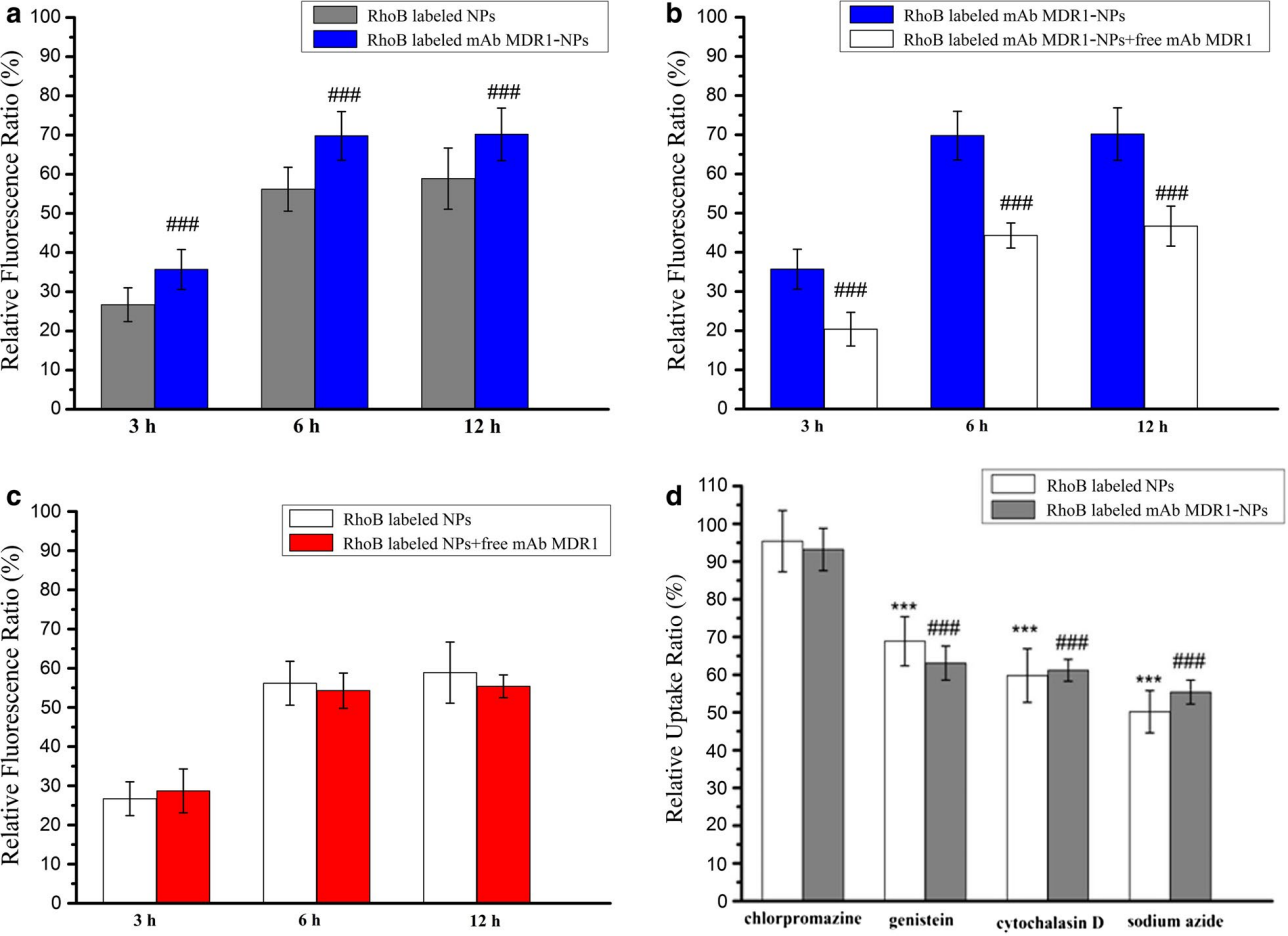
Analysis of the uptake mechanism of nanoparticles (NPs) in SMMC-7721/gefitinib cells. **a** Fluorescence spectrum analysis of the uptake of RhoB -labeled NPs and RhoB-labeled mAb MDR1-NPs in SMMC-7721/gefitinib cells. ^###^P < 0.001, vs RhoB-labeled NPs at different time interval. **b** Fluorescence spectrum analysis of the uptake of RhoB-labeled mAb MDR1-NPs in gefitinib-resistant cells with or without free mAb MDR1. ^###^P < 0.001, vs RhoB-labeled mAb MDR1-NPs in gefitinib-resistant cells without free mAb MDR1 at different time interval. **c** Fluorescence spectrum analysis of the uptake of RhoB -labeled NPs in gefitinib-resistant cells with or without free mAb MDR1. **d** Effects of endocytic inhibitors on the uptake ability of the two NPs in SMMC-7721/gefitinib cells. ***P < 0.001, vs RhoB-labeled NPs treated with chlorpromazine. ^###^P < 0.001, vs the RhoB-labeled mAb MDR1-NPs treated with chlorpromazine. The results of all experiments were expressed as mean ± SD (n = 3)

The relative fluorescence ratio (RFR, %) of NPs was quantified by calculating the percentage ratio of fluorescence intensity emitted by the internalized RhoB-labeled NPs to the initial fluorescence intensity from the total added RhoB-labeled NPs. It can be seen from Fig. 3 that both types of NPs depended on time variations in order to become internalized into the cells; moreover, the majority of both NPs completed their internalization within 6 h. The RFR (%) of both CS NPs and mAb MDR1-NPs had increased gradually, from approximately 26.7 and 35.7%, respectively, in the initial 3 h to over 56.2 and 69.8% at 6 h and, finally, they had slightly enhanced to 58.9 and 70.2% at 12 h (Fig. 3a). This indicated that when compared with unconjugated CS NPs, mAb MDR1-NPs showed higher uptake rates with the mediation of MDR1. Interestingly, when the combination of free mAb MDR1 and NPs were added to treat the cells, free mAb MDR1 competed with mAb MDR1-NPs to block MDR1 receptors on the cell surface. Therefore, the endocytosis of mAb MDR1-NPs was significantly inhibited and the RFR (%) of mAb MDR1-NPs at 6 h had decreased from 69.8 to 44.3% (Fig. 3b). On the contrary, there was no obvious variation in the internalization efficiency of CS NPs when cells were treated with free mAb MDR1 and non-mAb MDR1 (Fig. 3c). This further proved that the uptake of mAb MDR1-NPs was enhanced via the interaction between mAb MDR1 and MDR1 receptors. During the endocytosis inhibition test, various endocytic pathways of NPs were investigated by using different endocytic inhibitors, including chlorpromazine for inhibiting clathrin-mediated uptake, genistein for blocking caveolae-mediated uptake, cytochalasin D for inhibiting macropinocytosis, and sodium azide as an energy inhibitor. The results shown in Fig. 3d demonstrated that both caveolae and macropinocytosis mainly mediated the endocytic pathways of both NPs. Compared with treatments with non-inhibitors, the relative uptake ratios in cells treated with genistein and cytochalasin D were 68.9 and 59.8% for CS NPs, and 63.1 and 61.2% for mAb MDR1-NPs, respectively. The uptake of both NPs was energy dependent, and the relative uptake ratios had decreased to 50.3% for CS NPs and 55.4% for mAb MDR1-NPs with the addition of the energy inhibitor, sodium azide. In addition, there was no obvious variation in the internalization efficiency of both NPs, irrespective of whether chlorpromazine (an inhibitor of clathrin-mediated uptake) was added or not.

### MTT assay

**Fig. 4:**
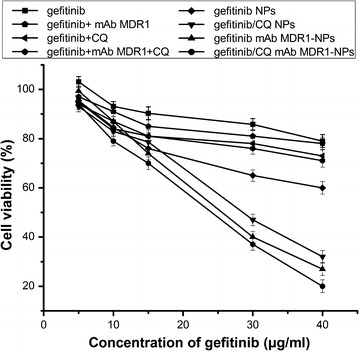
Viability of SMMC-7721/gefitinib cells after incubation with different preparations for 24 h. Data are presented as mean ± SD (n = 3)

An MTT assay was used to determine the effect of gefitinib on the proliferation of SMMC-7721/gefitinib cells. SMMC-7721/gefitinib cells were treated with different preparations containing different doses of gefitinib for 24 h. The MTT results (Fig. 4) showed that the cell viability of free gefitinib was not significantly decreased at all concentrations, ranging from 5 to 40 μg/mL, and more than 80% of cells survived. As free mAb MDR1 was mediated to block MDR1 and free CQ was used to inhibit the autophagy effect, the cytotoxicity of gefitinib was enhanced. This indicated that acquired drug resistance might be associated with higher levels of protective autophagy and the overexpression of MDR1 proteins. When compared with cell treatment featuring the simple mixing of gefitinib, mAb MDR1, and CQ, gefitinib/CQ mAb MDR1-NPs induced the strongest cell inhibition effects; furthermore, the half maximal inhibitory concentration (IC50) value of gefitinib/CQ mAb MDR1-NPs-treated, SMMC-7721/gefitinib cells was 22.3 μg/mL within 24 h. This finding indicated that the mAb MDR1-NPs, which specifically bound to the MDR1 receptor, had overexpressed at the drug-resistant cell surface and prevented gefitinib (a hydrophobic drug) from efflux outside the cells, thus improving the drug concentration at the tumor site. CQ, as an inhibitor of autophagolysosome formation, could inhibit autophagy in the resistant cells and the co-delivery of CQ and gefitinib by NPs showed higher cytotoxic rates. The mAb MDR1-NPs enhanced targeted drug delivery by effectively promoting drug accumulation in those cancer cells in which MDR1 was highly expressed on the cell surface by specific binding between mAb-MDR1 and MDR1 on the cell surface. Furthermore, by inhibiting autophagy and blocking the activity of the MDR1 receptor, the co-delivery of CQ and gefitinib, as well as the mediation of mAb MDR1-NPs, synergistically exerted a positive role on reversing the sensitivity of gefitinib within the cells and promoted the efficacy of chemotherapeutics.

### Cell apoptosis and necrosis

**Fig. 5:**
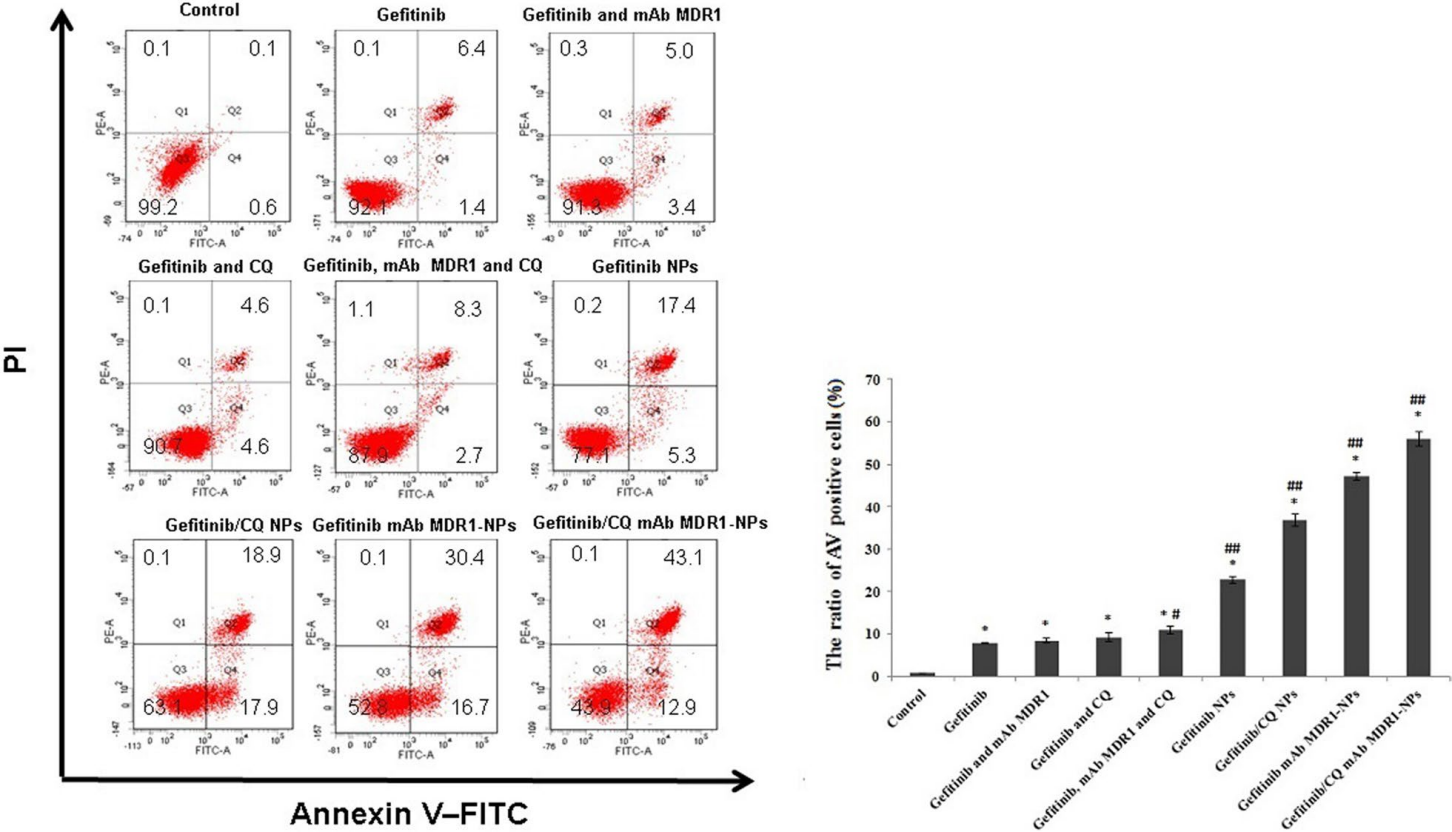
Flow cytometer analyses of the apoptotic and necrotic cells after 24 h’ incubation with different preparations. *P < 0.05, vs the ratio of AV positive cells in controlled group, ^#^P < 0.05, vs the ratio of AV positive cells in free gefitinib group, ^##^P < 0.01, vs the ratio of AV positive cells in free gefitinib group. Results are expressed as mean ± SD (n = 3)

The AV–FITC/PI staining assay also confirmed the findings obtained by the MTT assay. Flow cytometry analysis (Fig. 5) revealed that after 24 h of incubation with the treatment of free gefitinib and a combination of free gefitinib, mAb MDR1, and CQ, the ratios of AV positive cells in SMMC-7721/Gefitinib cells were very low, valued at 7.8% for gefitinib, 8.4% for gefitinib and mAb MDR1, 9.2% for gefitinib and CQ, and 11.0% for the combination of gefitinib, CQ, and mAb MDR1. This suggested that compared with free gefitinib, the added CQ and mAb MDR1 acted as potential tumor cell inhibitors which played a role in increasing the cells’ sensitivity to the apoptosis signal, but for the degradation of mAb MDR1 by the enzyme and the difficult uptake of CQ by passive diffusion, it was difficult to significantly improve cell apoptosis in the free state. When gefitinib was loaded into the CS NPs and acted on cells, the apoptotic rate increased to 22.7%, and positively charged CS NPs had combined with the negatively charged membrane, thus accelerating the internalization of NPs, enhancing the concentration of gefitinib at the tumor site, and increasing the apoptosis rate of SMMC-7721/gefitinib cells. When mAb MDR1 had bound to the surface of the NPs, the sensitivity of gefitinib to SMMC-7721/gefitinib cells was further enhanced and the ratio of AV positive cells treated with gefitinib mAb MDR1-NPs was 47.1%. Upon co-delivery of gefitinib and CQ in mAb MDR1-NPs, more NPs were transported into those cancer cells expressing MDR1 at high levels due to the interaction between mAb MDR1 and its receptors. Based on the specific binding between MDR1 and mAb MDR1, the suppression of MDR1 could not give play to transport the drugs out of the cells, thus reversed drug resistance and improved cell apoptosis. Autophagy was inhibited in the presence of CQ-loaded NPs and cell viability had further decreased. In this context, the greatest effect of cellular apoptosis was induced and the ratio of AV positive cells treated with gefitinib/CQ mAb MDR1-NPs was 56.0%.

### Western blot assay

**Fig. 6:**
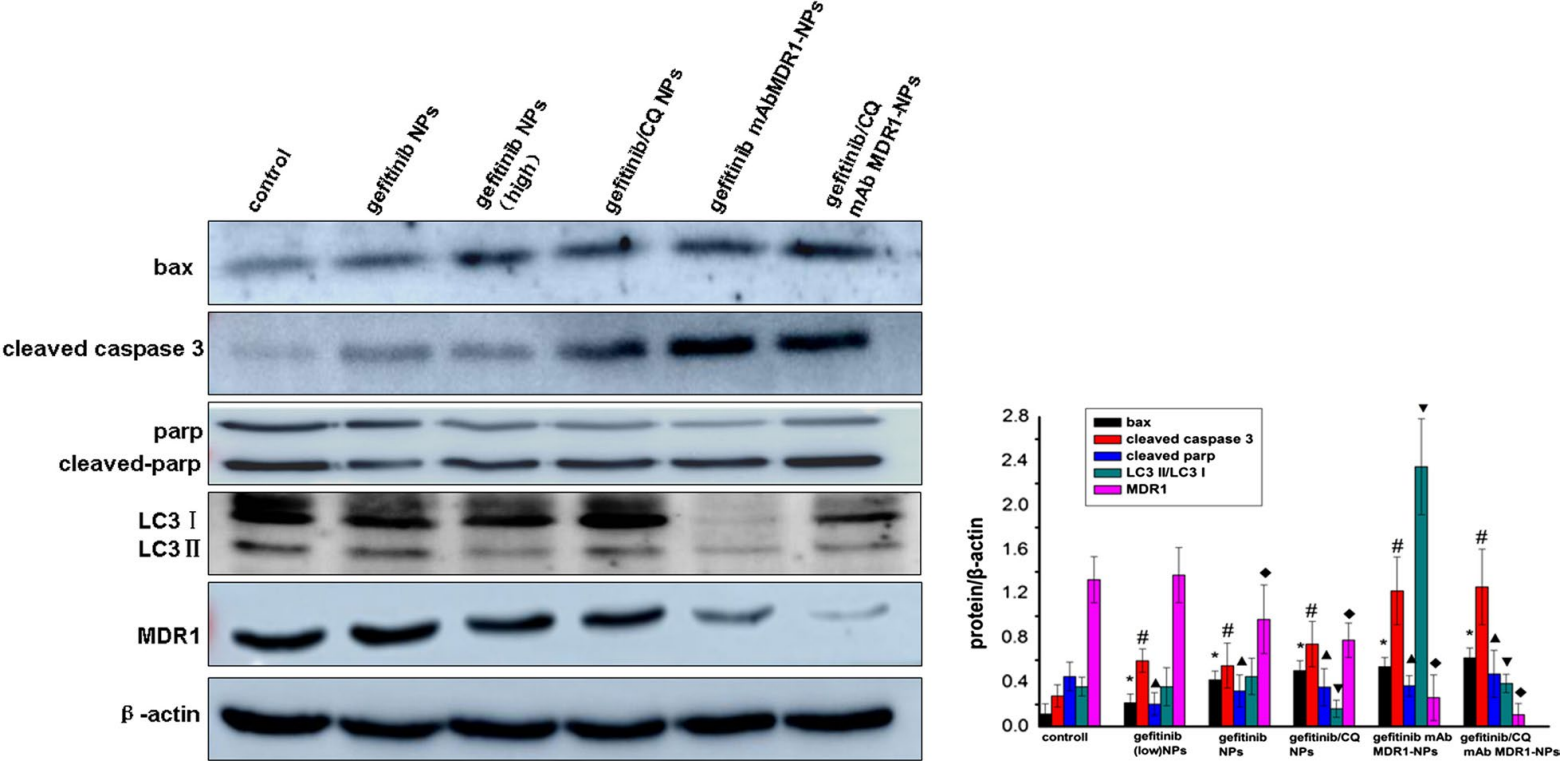
Western blot analyses of the expression levels of bax, cleaved caspase 3, Parp, LC3 and MDR1 proteins in cells after treatments. ^*^P < 0.05, vs the bax protein expression of controlled group, ^#^P < 0.05, vs the cleaved caspase 3 protein expression of controlled group, ^▲^P < 0.05, vs the cleaved parp expression of controlled group, ^▼^P < 0.05, vs the LC3 II/LC I expression of controlled group, ^◆^P < 0.05, vs the MDR1 expression of controlled group, data were presented as mean ± SD (n = 3)

The results, as shown in Fig. 6, highlighted that when compared with the control group, gefitinib induced cell apoptosis by upregulating the expression of bax, cleaved caspase 3. These findings indicated that with the mediation of gefitinib, the pro-apoptotic protein bax was first activated and transported to the mitochondrial membrane to form homodimers or multidimers. Finally, the cleaved caspase 3 was further activated, thus initiating caspase-dependent apoptosis of the tumor cells. Under the interaction between the mAb MDR1 and MDR1 receptors, the expression of the MDR1 protein was significantly downregulated, and the activity of apoptosis-related proteins (such as bax, cleaved caspase 3, and cleaved parp) was significantly enhanced. This indicated that gefitinib NPs might be internalized via MDR1-mediated uptake and the blocking of MDR1 increased the intracellular distribution and concentration of drugs, leading to the improvement of cytotoxicity. In addition, the delivery of CQ in mAb MDR1-NPs inhibited the occurrence of protective autophagy by lowing the ratio of LC3 II to LC3 I, and it further induced the highest expression of cleaved caspase-3 in SMMC-7721/gefitinib cells, indicating that the cells’ sensitivity to gefitinib was significantly enhanced through the blocking of MDR1 and the inhibition of autophagy, ultimately inducing significant cellular apoptosis effects. Interestingly, we preliminarily investigated the relationship between autophagy and the MDR1 protein. The results showed that the expression of the MDR1 protein was obviously downregulated, as autophagy was inhibited through the addition of CQ. This finding implied that CQ might be related to the role played by MDR1 in the development of acquired resistance. Moreover, CQ had inhibited the ATP-binding cassette (ABC) family of proteins, which play an important role in drug transportation and could reverse P-gp-mediated MDR (multiple drug resistance) in tumor cells [29, 30]. Taken together, autophagy and MDR1 might be the targets required to overcome acquired resistance in SMMC-7721/gefitinib cells, and their clinical use for inhibiting autophagy and MDR1 might represent some of the important strategies needed to overcome acquired resistance in SMMC-7721/gefitinib cells.

## Discussion

Gefitinib/CQ mAb MDR1-NPs were successfully prepared and characterized by their smaller particle size, positive zeta potential, higher drug encapsulation, and controllable release pattern. With the mediation of MDR1 receptors overexpressed on the cell surface as a specific targeting site, mAb MDR1 located at the surface of the NPs quickly bound to the MDR1 receptor to promote the internalization of NPs and to enhance the intracellular concentration of the drugs. The RFR (%) of both the CS NPs and mAb MDR1-NPs had increased gradually from approximately 26.7 and 35.7%, respectively, in the initial 3 h to over 56.2 and 69.8% at 6 h. Furthermore, by blocking MDR1 by mAb MDR1, the activity of MDR1 was inhibited and the efflux of the drug via MDR1 was reduced; therefore, gefitinib/CQ mAb MDR1-NPs greatly facilitated gefitinib uptake by effectively transporting NPs into the cells. The results further confirmed that as autophagy was inhibited by downregulating the ratio of LC3 II and LC3 I in the presence of CQ-loaded NPs, cell viability was significantly decreased further, and the greatest cell apoptosis effects were induced through the efficient prohibition of autophagy. Taken together, gefitinib/CQ mAb MDR1-NPs induced the strongest cell inhibition effects. The IC50 value in gefitinib/CQ mAb MDR1-NPs-treated, SMMC-7721/gefitinib cells within 24 h was 22.3 μg/mL and the ratio of AV positive cells treated with gefitinib/CQ mAb MDR1-NPs was 56.0%. Western blot assay was also performed to determine the level of related proteins to clarify the role of autophagy and the expression of MDR1 proteins on overcoming acquired drug resistance. The results showed that after being treated with gefitinib/CQ mAb MDR1-NPs, mAb MDR1 efficiently suppressed the expression of the MDR1 protein, while CQ significantly inhibited autophagy by downregulating the ratio of LC3 II and LC3 I; therefore, the expression of apoptosis-related proteins such as bax, cleaved caspase-3, and cleaved parp was significantly up-regulated. All of these results showed that a higher level of autophagy—as a protective mechanism—and the overexpression of MDR1 could participate in the induction of tumor-acquired resistance in tumor cells. To determine whether the overexpression of MDR1 and the augmentation of autophagy contributed to cell death, we manipulated autophagic activity using an autophagy inhibitor (CQ) and mAb MDR1 targeting MDR1. We found that the reversal activity of gefitinib-acquired resistance was significantly exacerbated in the presence of CQ, which inhibited autophagy and blocked the expression of MDR1. These results suggested that CQ was capable of reversing MDR; the induction of autophagy represented a defense mechanism, and inhibiting this process may be an effective strategy to augment the reversal activity on overcoming acquired EGFR-TKI resistance. In addition, mAb MDR1 greatly facilitated gefitinib uptake by effectively transporting NPs into cells and enhancing the cytotoxic effect in SMMC-7721/gefitinib cells upon mediation of mAb MDR1 to suppress MDR1 expression.

## Conclusions

Briefly, we found that mAb MDR1-modified CS NPs, when combined with the co-delivery of gefitinib and CQ, showed targeting and therapeutic potential on enhancing the delivery of anticancer drugs and inducing significant cell apoptosis against acquired EGFR-TKI resistance through the modulation of autophagy and blocking the activity of MDR1. This finding implied that autophagy and MDR1 may serve as dual targets to overcome acquired resistance in SMMC-7721/gefitinib cells, and the clinical application of autophagy and MDR1 inhibition might be one of the important strategies for overcoming acquired EGFR-TKI resistance in SMMC-7721/gefitinib cells.

## Abbreviations

TKIs: tyrosine kinase inhibitors
EGFR: epithelial growth factor receptor
CS: chitosan
NPs: nanoparticles
CQ: chloroquine
